# RNA m6A Methylation Regulators Multi-Omics Analysis in Prostate Cancer

**DOI:** 10.3389/fgene.2021.768041

**Published:** 2021-11-26

**Authors:** Hao Su, Yutao Wang, Hongjun Li

**Affiliations:** ^1^ Department of Urology, Chinese Academy of Medical Sciences, Peking Union Medical College, Peking Union Medical College Hospital, Beijing, China; ^2^ Department of Urology, The First Affiliated Hospital of China Medical University, Shenyang, China

**Keywords:** m6A methylation, muti-omics analysis, prostate cancer, vitro experiment., methylation prognosis model

## Abstract

RNA N6-methyladenosine (m6A) methylation is known to be the most popular RNA modification in animals. Many research reports have elaborated on the effects of m6A regulators in medical practice, such as diagnosis, prognosis, and treatment. M6A modification has evident impacts on many aspects of RNA metabolism, just like RNA splicing, processing, translation, and stability. M6A also has a magnificent role in numerous types of cancers. We analyzed the prostate cancer datasets, from The Cancer Genome Atlas (TCGA) database, for every recognized m6A regulator in their gene expression, DNA methylation status and copy number variations (CNVs). We also systematically analyzed the relationship between different m6A regulators and the prognosis of prostate cancer. The results illustrated considerable differences in the expression of various m6A regulators between the prostate and normal cancer samples. At the same time, there were evident differences in the expression of various m6A regulators in prostate cancers with different Gleason scores. Subsequently, we determined CBLL1, FTO, YTHDC1, HNRNPA2B1 as crucial m6A regulators of prostate cancer. Premised on the expression of CBLL1, we also identified potential therapeutic agents for prostate cancer, and knockdown of FTO prominently inhibited prostate cells migration and invasion *in vitro* experiment.

## Introduction

Prostate cancer (PCa) is the most commonly occurring malignant tumor of the male genitourinary system and ranks second among the common malignant tumors in men worldwide. Its incidence rate is highest in the United States (Siegel et al., 2021). It had around 1.1 million diagnoses in 2012 worldwide, which accounted for 15% of the diagnosed malignant tumors (Ferlay et al., 2015). Androgen deprivation therapy (ADT) is the standard therapy used in treating patients with advanced prostate cancer. Nevertheless, the majority of advanced prostate cancers ultimately develop into castration-resistant prostate cancer (CRPC) that has dismal prognosis (Martínez-Breijo et al., 2018). Despite the recent development of novel treatments, patients with advanced PCa have a high death rate (McNevin et al., 2021). Identifying appropriate biomarkers is essential for the early diagnosis, treatment, and prognosis of prostate cancer. Histological grading of prostate cancer is an important parameter for predicting the treatment and prognosis response. To date, Gleason grading forms the basis of prostate cancer grading (Egevad et al., 2019). The Gleason grade is assigned based on the architecture and arrangement of the malignant cells within the tumor. The Gleason grades are used to generate a Gleason score, which ranges from 2 to 10. The Gleason score of 7 indicates medium-risk prostate cancer, less than 7 indicates low-risk prostate cancer, whereas the Gleason score of more than 7 is indicative of high-risk prostate cancer (Epstein et al., 2016a). Currently, the minimum score of the Gleason system is specified as 6, although it ranges from 2 to 10. This leads to unnecessary treatment in some patients (Epstein et al., 2016b). Hence, the Gleason score is important in determining the prognosis of prostatic malignancies. However, it has many drawbacks, which have an impact on the treatment and outcome of patients. Therefore, the grading of prostate cancer by Gleason score is not perfect. Thus, more effective classification methods are needed to grade prostate cancer.

N6-methyladenosine (m6A) RNA modification is an imperative mechanism of post-transcriptional regulation of gene expression. m6A acts as the most predominant as well as conserved internal mRNA modification and accounts for >80% of all types of RNA methylation modifications. It has a crucial function in regulating biological processes, including embryonic development and reproduction (Zheng et al., 2020). m6A regulators are of 3 types: writers (m6A methyltransferases), erasers (demethylases), and readers (recognize m6A). m6A methyltransferases/writers catalyze m6A. The core components of the writer are RBM15/15B, WTAP, METTL3, METTL14, VIRMA, and ZC3H13. However, since m6A modification is both dynamic and reversible, it can be reversed by demethylases/erasers. Erasers such as ALKBH5 and FTO maintain the balance of m6A modification in the transcriptome. Besides writers and erasers, m6A selectively binds m6A binding proteins, also called “readers.” Representative readers are IGF2BP1/2/3, eIF3, HNRNP, YTHDC1/2, and YTHDF1/2/3(Wang T. et al., 2020). Evidence suggests the function of m6A modification in regulating RNA metabolism, RNA folding and structure, nuclear processing and mRNA export, mRNA maturation, degradation, and translation (Zhao et al., 2017). RNA m6A serves as a novel epigenetic regulatory mechanism in several biological processes such as circadian rhythm, embryogenesis, sex determination, adipogenesis, heart rate, stress response, and neurodevelopment. m6A affects these biological processes and molecular functions either by promoting or inhibiting the expression of target genes (Luo et al., 2018).

Bioinformatics analysis is a convenient tool to identify the m6A regulatory factors that are suitable for tumor classification and prognosis of various types of cancers. Evidence indicates the association of m6A modification with tumor proliferation, differentiation, invasion, and metastasis in many malignant tumors like glioblastoma, breast cancer, liver cancer, acute myeloid leukemia, lung cancer, colorectal cancer and urological tumors, such as prostate cancer, renal cell carcinoma, bladder cancer, Wilms tumor and testicular germ cell tumors (Chen et al., 2019; Li Y. et al., 2020). With further expansion of research, we will see the magnificent impact of m6A on cancer cell proliferation.

In this research, we analyze the molecular alterations of m6A regulatory factors as well as their unique characteristics in prostate cancer.

## Materials and Methods

### Data Collection

Prostate cancer datasets from The Cancer Genome Atlas (TCGA) were accessed utilizing the UCSC Xena platform (Goldman et al., 2020). The obtained gene expression profiles were processed from the Illumina HiSeq 2000 (HiSeq) platform and then converted into log2 (RSEM+1) format. The data of somatic mutation was collected in Mutation Annotation Format (MAF). The experimental measurement of gene-level copy number variations (CNVs) was done utilizing the Affymetrix Genome-Wide Human SNP Array 6.0 platform and preprocessed utilizing the GISTIC2 method (Mermel et al., 2011). Measurement of DNA methylation levels approximated by beta (β) values was done premised on the GPL13534 platform (Illumina Infinium Human Methylation 450 Bead-Chip array). The DNA methylation *β*-values are continuous variables ranging between 0 and 1 and represent methylated alleles’ proportion. The data of miRNA expression were processed from the Illumina HiSeq 2000 platform, whereas the miRNA-target interactions were accessed from miRTarBase dataset (Chou et al., 2018). Information on the subtype and survival of each sample was contained in the phenotype data.

### Correlation Analysis

The Pearson correlation coefficients between gene expression, DNA methylation, CNVs, or miRNA expression, respectively, were calculated in the R package using the cor. test function. The DNA methylation probes included in the analysis are only those with missing values in lower than 50 percent of samples. The miRBaseVersions.db R package was utilized to convert distinct varieties of miRNA IDs (Haunsberger et al., 2017). It is important to determine prostate cancer m6A regulators. A Random Forest algorithm premised on gene expression levels was performed to assess the importance of m6A regulators in differentiating various types of prostate cancers. This procedure was processed in R with the Random Forest package (Kimura et al., 2019). Additionally, the varSelRF R package was utilized to determine variable selection (Diaz-Uriarte, 2007).

### Sample Clustering Analysis

Unsupervised hierarchical clustering analysis was executed with filtered DNA methylation probes, whose *β*-values fit the criteria described below: 1) the absolute value of Pearson correlation coefficient with gene expression was over 0.4 2) the standard deviation (SD) amongst all the samples was over 0.2. To determine the sum of clusters for basal-like and luminal samples, consensus clustering was done utilizing the ConsensusClusterPlus package in R by resampling iteration(80% resampling rate, 50 iterations) (Wilkerson and Hayes, 2010). The cluster number was identified based on the relative change in area under the cumulative distribution function (CDF) curve. The pheatmap R package was utilized to generate the heatmap corresponding to the consensus clustering.

### Differential Expression Genes Analysis

DEGs between group 1 and group 3 samples were identified *via* the DESeq2 R package (Love et al., 2014). In summary, the initial log2 (RSEM+1) values were converted into RSEM values, which were then grounded to integers. Subsequently, the DESeqDataSet from the Matrix function was utilized to import the expression matrix. The genes that fit the set criteria of adjusted *p*-value <0.05 and fold change <0.66 or >1.5 were deemed as DEGs between group 1 and group 3 samples.

### Functional Enrichment Analysis

Kyoto Encyclopedia of Genes and Genomes (KEGG) pathway enrichment analysis of the DEGs between group 1 and group 3 samples were executed utilizing clusterProfiler R package (Yu et al., 2012). The marker genes for each immune cell population were curated from existing research. The relative abundance of various kinds of Tumor-infiltrating lymphocytes (TILs) in each sample was examined *via* single-sample gene set enrichment analysis (ssGSEA) utilizing the GSVA R package.

### Least Absolute Shrinkage and Selection Operator Regression

The method is a compressed estimation. It constructs a more refined model *via* constructing a penalty function that compresses some coefficients and sets some coefficients to zero. As a result, it keeps the advantage of subset contraction and is a biased estimator for complex collinear data. In this study, we used LASSO to construct a prognostic model of M6A regulators methylation sites.

### Drug Sensitivity Analysis

Data on the drug sensitivity of cancer cell lines (CCLs) were extracted from the PRISM Repurposing database (19Q4, released December 2019) and Cancer Therapeutics Response Portal (CTRP v.2.0, released October 2015). The PRISM comprises the sensitivity data for 1448 compounds over 482 CCLs, whereas the CTRP covers the sensitivity data for 481 compounds over 835 CCLs. Both of these two databases depict the area under the dose-response curve (area under the curve—AUC) values, as a measure of drug sensitivity, with smaller AUC values illustrating greater sensitivity to therapy. The missing AUC values were imputed utilizing K-nearest neighbor (k-NN) imputation. Prior to imputation, we excluded compounds that had over 20% of missing data. Considering that the CCLs in both datasets were acquired from the CCLE project, molecular data in CCLE were then used for subsequent CTRP and PRISM analyses.

### Cell Culture and Transfection

The human prostate cancer cell lines, DU145 and PC3, were purchased from the National Collection of Authenticated Cell Cultures (Shanghai, China). DU145 and PC3 were cultured in RPMI 1640 with 10% serum and incubated at 37°C, 5% CO2. The two Small interfering RNAs (JTSBIO) sequences used to reduce FTO expression sequences used to reduce FTO expression were as follows: GCAGUGUAUCUGAGGAGCUCCAUAA UUAUGGAGCUCCUCAGAUACACUGC; CAGGCUGCACCUACAAGUACCUGAA UUCAGGUACUUGUAGGUGCAGCCUG.

### RNA Extraction and Quantitative Real-Time PCR

Total RNA from cells were extracted with RNAiso Plus (Takara Biotechnology, Dalian, China) and then reverse transcribed into cDNA with Prime Script RT Master Mix (Takara Biotechnology, Dalian, China). RT-qPCR was performed using SYBR Premix EX Taq™ (Takara) and 2^-ΔΔCT^ method was used to analyze the expression level of FTO normalized to GAPDH. The primer sequences are as follows: FTO (forward: ACTTGGCTCCCTTATCTGACC; reverse: TGTGCAGTGTGAGAAAGGCTT) and GAPDH (forward: GGAGCGAGATCCCTCCAAAAT; reverse: GGCTGTTGTCATACTTCTCATGG).

### Transwell Assay

Transwell chambers with 8-μm pores (Corning Costar, Corning, NY, USA) placed in 24-well plates were used to detect the ability of cells to migrate and invade.50% Chambers with or without Matrigel (BD, San Diego, CA, USA) was used to detect the cell invasiveness or migration. 600 μL of RPMI 1640 supplemented with 10% serum was added below the chamber, and 200 μL of serum-free RPMI 1640 containing cells was added above the chamber. After incubation for 24 h at 37°C in an atmosphere of 5% CO2, the chambers were placed into a 1.0% crystal violet. After 20 min, 1.0% crystal violet was washed with phosphate-buffered saline. Photographs were observed and taken with a microscope, and the number of cells passing through the chambers was counted with ImageJ.

### Wound-Healing Migration Assay

Cells were seeded into 6-well plates. When 90–100% confluence of the 6-well plate was achieved, a linear scratch was made with a 1000-µL sterile pipette. and cells were incubated with serum-free RPMI 1640. Photographs were taken with a microscope at 0 and 48 h. The migration ability of the cells was observed by comparing the width of the scratches in the photographs at the two time points.

### Statistical Analysis

All analyses were carried out utilizing the R computing framework. The Wilcoxon rank-sum test was utilized to contrast the differences in m6A regulators expression between prostate tumor and control samples. Subsequently, Kruskal-Wallis analysis was executed to contrast the gene expression among different subtypes of prostate cancer. Univariate Cox proportional hazards regression analysis was executed to assess the relationship between DNA methylation level, gene expression level, survival time, and CNV utilizing the coxph function with survival R package (Sinnott and Cai, 2016; Sarkar et al., 2017). Log-rank tests, as well as Kaplan-Meier survival analyses, were conducted to contrast the survival time of two clusters, which was processed with the survival R package. The time-dependent area under the receiver operating characteristic curve (AUC) was computed using the timeROCR package.

## Results

### Alterations of m6A Regulators in Prostate Cancer

**FIGURE 1:**
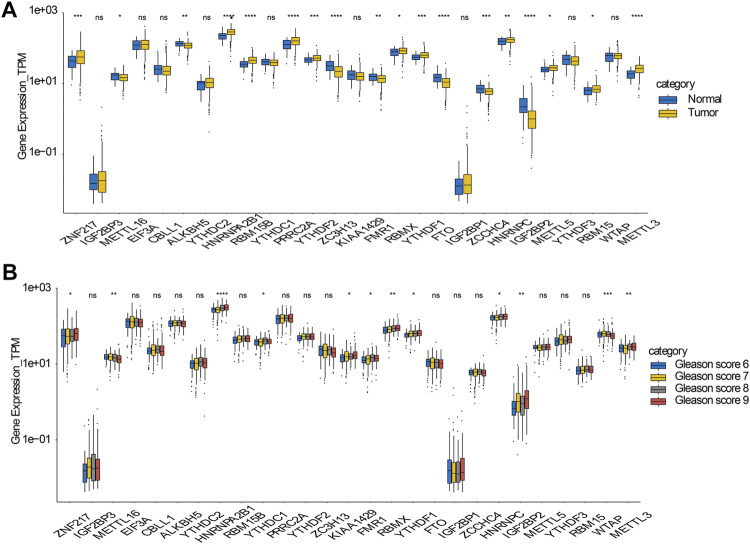
Expressions and genetic variations of m6A regulators in prostate cancer **(A)** Boxplot showing the m6A regulators with highly significant difference in their RNA expression between normal and tumor samples **(B)** Boxplot showing the m6A regulators with highly significant difference in prostate cancers with different Gleason scores.

The raw data and R code were uploaded in Supplementary file. The analysis strategy of this paper was shown in flow chart. Existing research has verified that abnormal expression of m6A regulators is linked to tumorigenesis and the progression of many cancers. Hence, we wanted to figure out whether this incidence can be detected in prostate cancer. Currently, 27 genes have been recognized as m6A regulators that act either directly or indirectly in functions such as m6A recognition, removal, or deposition. Initially, we scrutinized and compared the expression levels of these m6A regulators between prostate cancer and normal samples. Of the 27 genes, 18 exhibited a significant differential expression between the normal and prostate cancer samples. This suggests their possible participation in tumorigenesis of prostate cancer (Figure 1). In addition, in prostate cancers with different Gleason scores (6–9), 12 genes showed significantly different expression levels (Figure 1).

### m6A Regulators Multi-Omics Prognostic Value in Prostate Cancer

**FIGURE 2:**
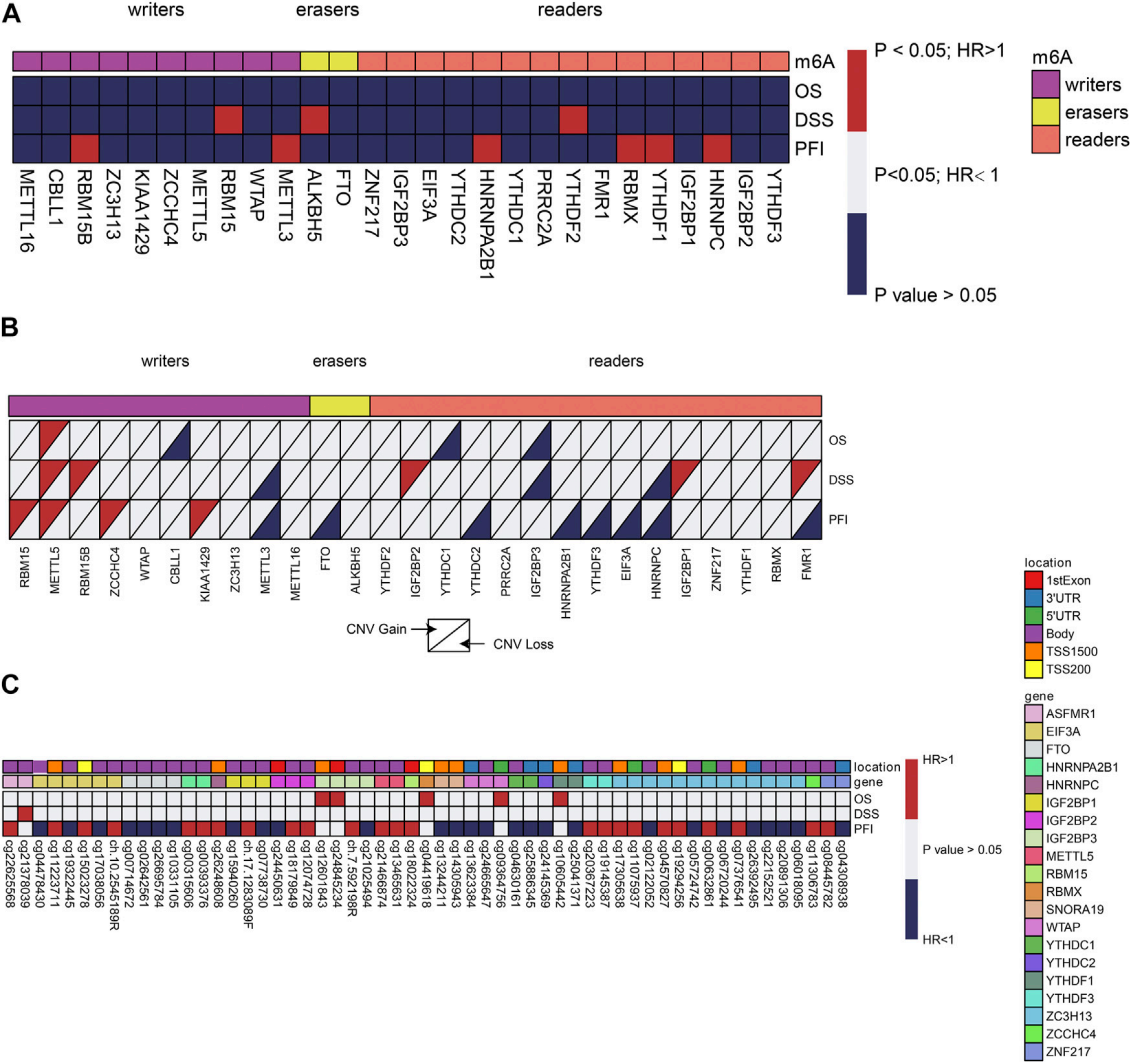
Univariate cox regression analysis of m6A regulators. **(A–C)** Univariate cox regression analysis of the association between overall survival (OS), disease-free survival (DFS), or progression-free survival (PFS) and gene expressions. Blue box, protective factors (HR < 1 and *p* < 0.05); Red box, risky factors (HR > 1 and *p* < 0.05); white box, *p* > 0.05. The sample size used in each cox regression analysis was marked in brackets.

Knowing the role of m6A regulators in the prognosis of different cancers, we pursued to examine their probable prognostic values in prostate cancer. Univariate Cox proportional hazards regression analysis was executed for gene expression level, CNV, and DNA methylation. With regards to gene expression, RBM15/15B, ALKBH5, HNRNPA2B1, HNRNPC, YTHDF1/2, METTL3, and RBMX seemed to be risk genes with Hazard Ratio (HR) > 1 (Figure 2A). Moreover, it was discovered that even CNVs of m6A regulators exhibited prognostic values. For instance, a copy number gain of other 9 m6A regulators worsened prognosis, whereas a copy number loss of FTO improved prognosis (Figure 2B). In reference to DNA methylation, we detected an aggregate of 57 CpG sites situated on 20 genes with DNA methylation levels that were linked to the Overall survival (OS), Disease-free survival (DFS), or progression-free survival (PFS) of prostate cancer patients (Figure 2C). The majority of these methylation sites displayed a protective function in prognosis. However, increased methylation levels of 16 CpG sites situated on RBM15B, YTHDF3, FMR1, WTAP, IGF2BP1/2/3, HNRNPA2B1, and eIF3A were linked to dismal prognosis.

### Copy Number Variations Perturb the m6A Regulator Expression in Prostate Cancer

**FIGURE 3:**
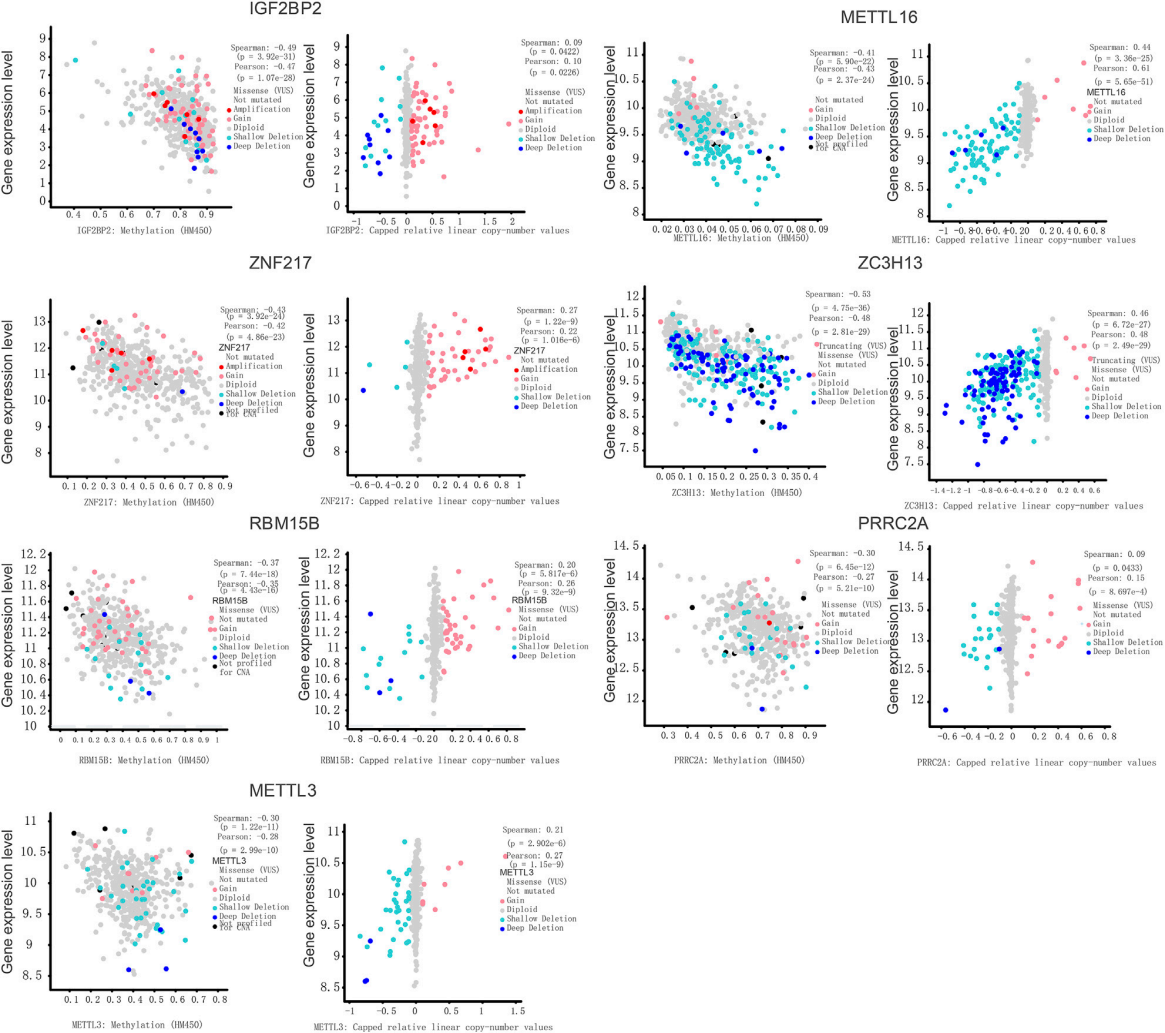
Copy-number values(CNV) of m6A regulators and capped relative linear CNVs.

Gene expression levels are known to be influenced by multi-layered genomic features that include miRNA expression, DNA mutation, DNA methylation, and CNV. For m6A regulators in prostate cancer, the abnormal regulatory elements were compared successively in prostate cancer against normal samples. To assess the potential influence of CNVs on gene expression, we conducted a correlation analysis between CNV and gene expression levels. Seven regulators, including IGF2BP2, METTL3, METTL16, ZNF217, ZC3H13, RBM15B, and PRRC2A, exhibited significant correlations between CNVs and gene expression levels in prostate tumors (Figure 3). This indicates that the CNVs of these seven genes could help to disturb their gene expression in the tumors. In this study, we offer an in-depth understanding of genetic, transcriptional, and post-transcriptional changes of 27 recognized m6A regulators in prostate cancer, which is suggestive of their probable functions in various regulatory mechanisms and tumorigenesis. Premised on changes in the DNA methylation, copy numbers, and gene expression, few genes were detected as possible predictors for the survival of patients. Sub-classification of prostate cancers was premised on DNA methylation of m6A regulators.

### Determine m6A Regulator Subtype With Different Prognostic Significance

**FIGURE 4:**
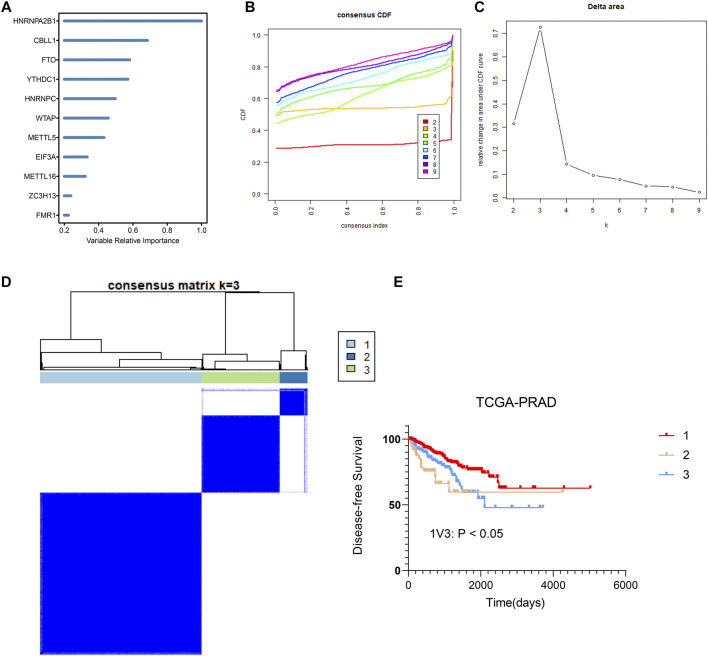
Identification and consensus clustering analysis of m6A regulators **(A)** Random forest analysis ranking the importance of m6A regulators in prostate cancer based on their gene expression levels. **(B,C)** Relative change in area under cumulative distribution function (CDF) curve based on results of consensus clustering for *k* = 2 to 9. **(D)** Consensus clustering matrix for *k* = 3. **(E)** Comparison of DFS between group 1、group 2 and group 3 samples in prostate cancer.

**FIGURE 5:**
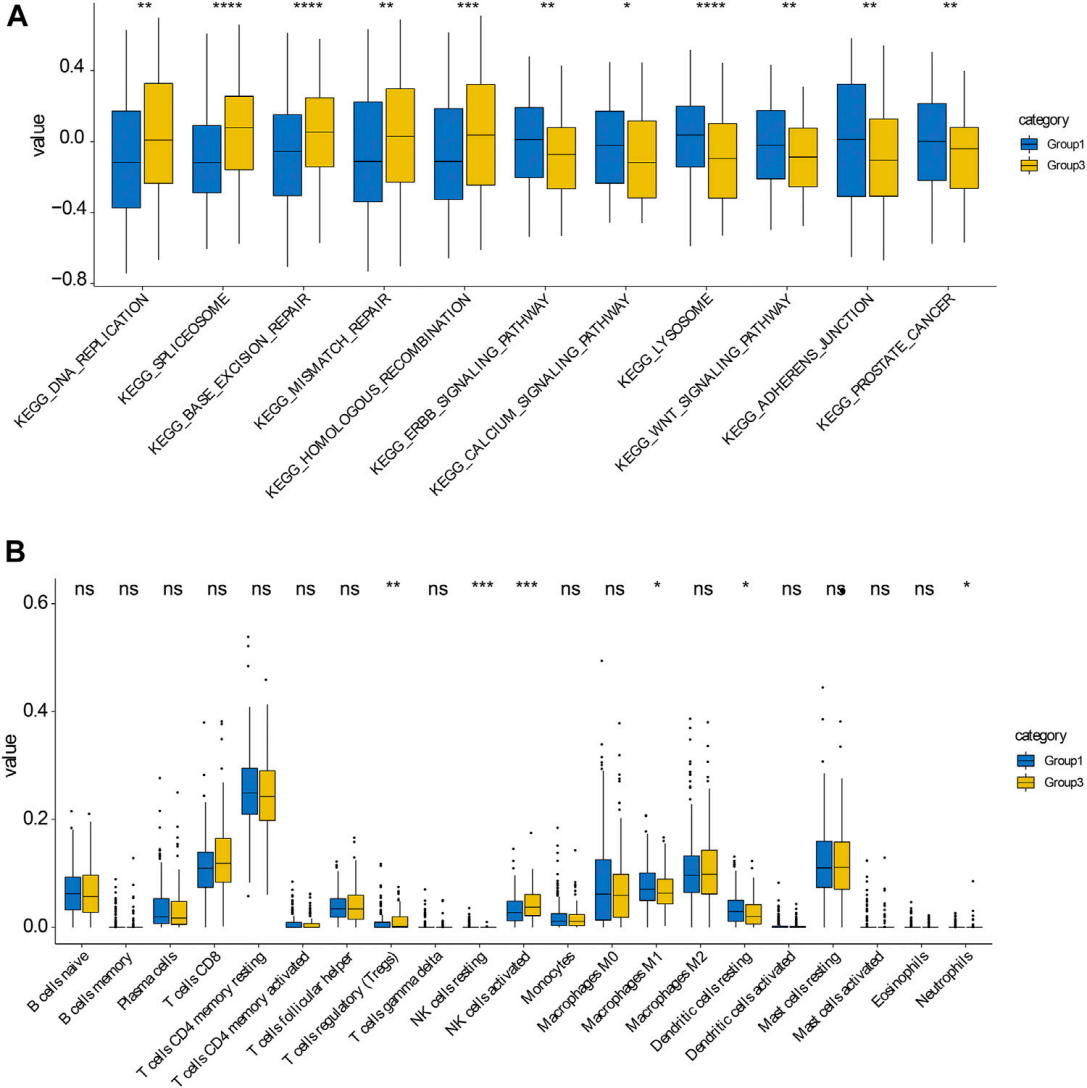
KEGG enrichment analysis in group 1 and group 3 subtypes **(A)** KEGG enrichment analysis of genes in group 1 and group 3 subtypes, respectively. **(B)** KEGG enrichment analysis of cells in group 1 and group 3 subtypes, respectively.

Premised on the gene expression of 27 regulators, random forest machine learning was employed to rank the gene importance and varSelRF method for selection of variables (Figure 4A). We determined the threshold based on importance >0.6. We determined four important genes, which are HNRNPA2B1, CBLL1. FTO, YTHDC1. The methylation site values of important genes were applied to consensus clustering analysis for different prostate cancers. Subsequently, consensus clustering was executed in R with the *β*-values of probes. *k* = 3 was considered to be the optimal outcome, with the clustering stability rising from *k* = 2 to *k* = 9 (Figures 4B,C). Therefore, we obtained three different prostate cancer subtypes, and the results showed that the dark blue within the subtype suggested strong correlation between samples within the group, while the white color was dominant between samples, suggesting weak relationship between different groups(Figure 4D). Notably, as per the clustering outcomes, the prostate cancers were efficaciously separated into three groups. The patients in different groups had different disease-free survival (DFS) (Figure 4E). Subsequently, KEGG functional enrichment analysis disclosed that genes in group 1 and group 3 subtypes showed great differences in expression (Figure 5A). These genes were involved in DNA replication, spliceosome, base excision repair, WNT signaling pathway, mismatch repair, homologous recombination, Erbb signaling pathway, calcium signaling pathway, lysosome, adherens junction, and prostate cancer. To further decipher differences between the two groups, we compared immune cell infiltration analysis of cells of group 1 and group 3 subtypes. These results also showed significant differences in follicular helper T cells, CD8^+^ T cells, CD4^+^ T cells, NK cells activated, macrophages M0, M1, M2, and regulatory T cells (Figure 5B). Overall, we identified the significance of m6A regulators in prostate cancer premised on their gene expression levels. We further utilized these expression patterns to successfully sub-classify the prostate cancers into three groups. KEGG functional enrichment analysis illustrated that genes in group 1 and group 3 subtypes showed great differences.

### Identification of Potential Therapeutic Agents for PCa

**FIGURE 6:**
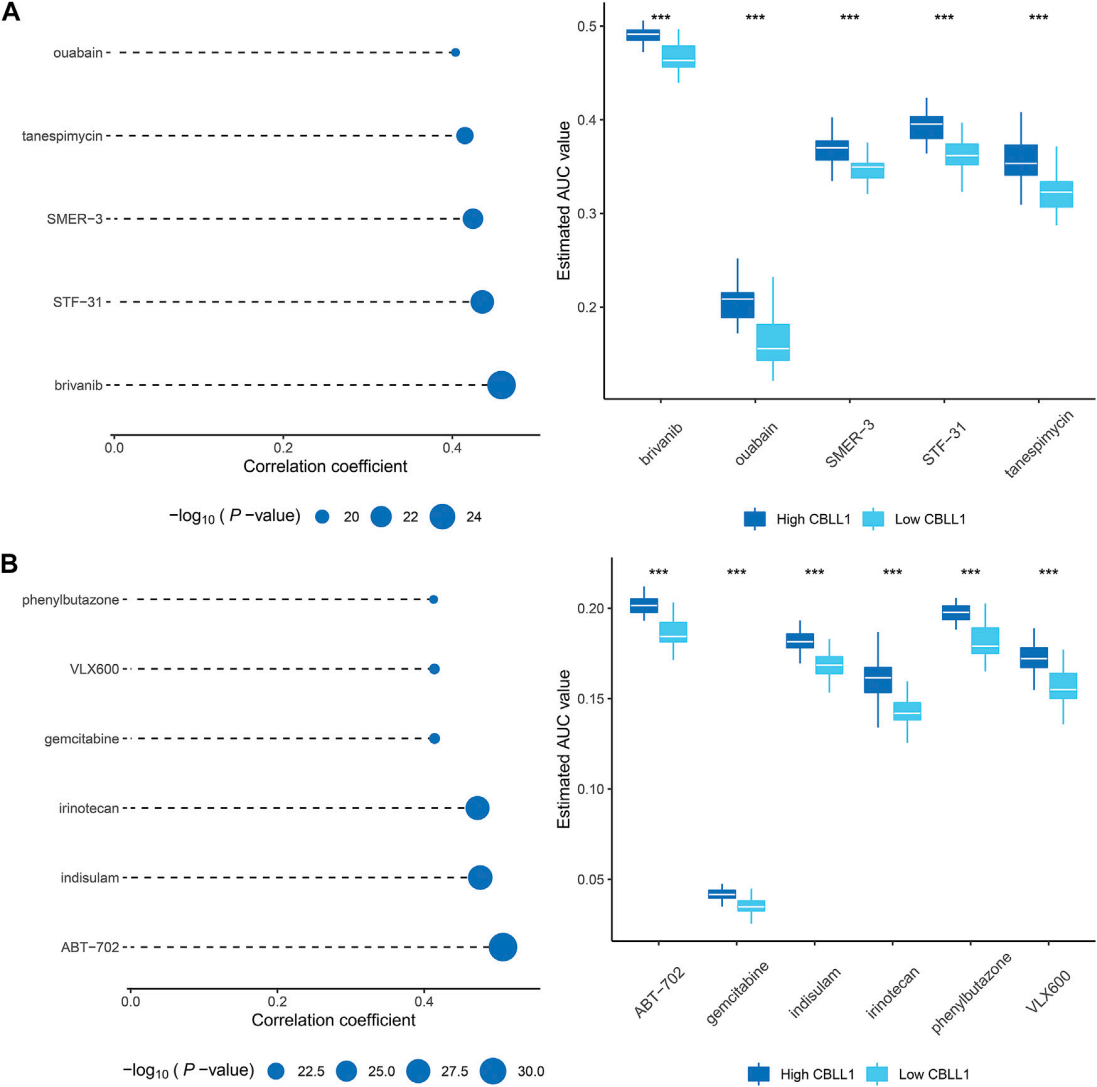
Identification of potential therapeutic agents for PCa **(A)** The results of Spearman’s correlation analysis and differential drug response analysis of five CTRP-derived compounds. **(B)** The results of Spearman’s correlation analysis and differential drug response analysis of six PRISM-derived compounds.

Compared with normal tissues, the content of CBLL1 in prostate cancer tissues was lower, and its expression has an adverse effect on the development and prognosis of prostate cancer. CBLL1 also has a superior ability to predict survival in prostate cancer patients. Drugs showing a high positive correlation with CBLL1 might have potential therapeutic effects in PCa patients. Therefore, in this research, premised on the expression of CBLL1 in PCa, we used two different methods to identify appropriate candidate agents with increased drug sensitivity in PCa patients. We used CTRP as well as PRISM-derived drug response data to do the analysis, respectively. First of all, we conducted differential drug response analysis between CBLL1 score-high (top decile) and CBLL1 score-low (bottom decile) to detect compounds that have higher approximated AUC values in the PCa cohort (log2FC > 0.10). Then, we conducted a spearman correlation analysis between AUC value and CBLL1 score to choose proper compounds with positive correlation coefficients (Spearman’s r < −0.30 for CTRP or−0.35 for PRISM). Through the above analysis, we found five CTRP-derived compounds that include brivanib, ouabain, SMER-3, STF-31, tanespimycin (Figure 6A). At the same time, we also found six PRISM-derived compounds that include ABT-702, gemcitabine, indisulam, irinotecan, phenylbutazone, VLX600 (Figure 6B). These compounds exhibited higher approximated AUC values in CBLL1 score-high cohort and a positive correlation with CBLL1. So, these compounds were thought to be potential therapeutic agents for PCa.

### M6A Regulator Genes Methylation Prognosis Model

**FIGURE 7:**
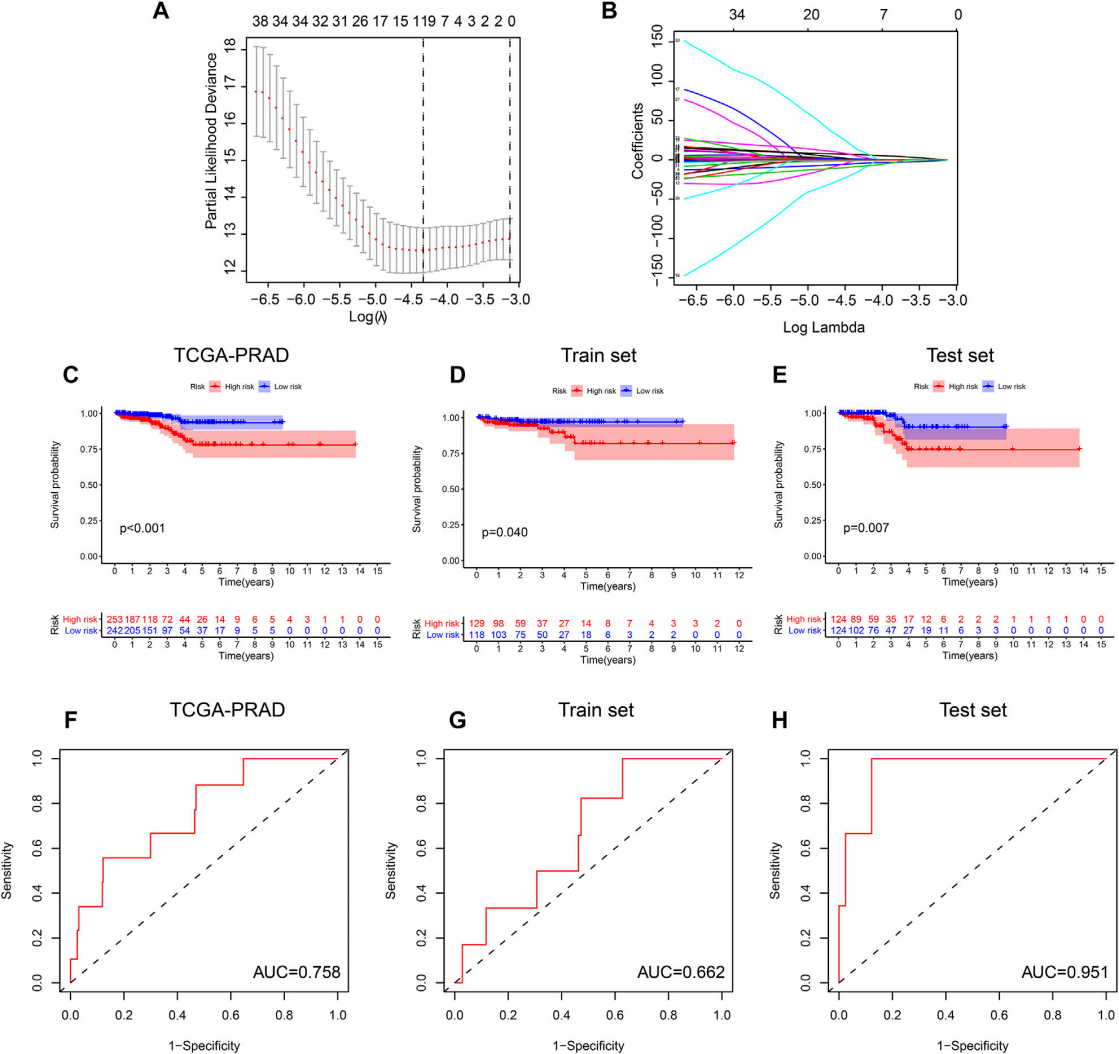
M6A regulator genes methylation prognosis model: **(A,B)** The process of Lasso regression was shown. **(C)** The prognostic model of the methylation level of the M6A regulator in the TCGA-PRAD cohort can significantly distinguish high- and low-risk patients (*p* < 0.001). **(D,E)** The classification statistics of the risk score in the training set is *p* = 0.04, the classification statistics of the risk score in the validation set is *p* = 0.007. (FGH) the AUC area of the risk score in the TCGA-PRAD cohort was 0.758. The AUC area in the training set cohort is 0.662, and the AUC area in the validation set cohort is 0.951.

Based on the results of random forest, we include the methylation sites of 6 regulators (HNRNPA2B1, CBLL1, FTO, YTHDC1, HNRNP and WTAP), the methylation site which bind gene importance is greater than 0.4 was taken into lasso regression. The process of Lasso regression was shown in Figures 7A,B, we constructed the M6A regulator genes methylation prognosis model: risk score = 13.27 * cg00315606–12.15 * cg00714672–49.80 * cg26692097. We found that the prognostic model of the methylation level of the M6A regulator in the TCGA-PRAD cohort can significantly distinguish high- and low-risk patients (*p* < 0.001) (Figure 7C). At the same time, in order to evaluate the predictive effect of the risk model, we randomly divide the TCGA - PRAD samples into two groups. The training set contains 247 samples and the validation set contains 248 samples. The classification statistics of the risk score in the training set is *p* = 0.04 (Figure 7D), *p* = 0.007 in the validation set (Figure 7E). Finally, we used the AUC parameters of the ROC experiment to test the predictive power of the model. The results showed that the AUC area of the risk score in the TCGA-PRAD cohort was 0.758. The AUC area in the training set cohort is 0.662, and the AUC area in the validation set cohort is 0.951 (Figures 7F,G,H).

### Knockdown of HNRNPA2B1 Significantly Inhibited Prostate Cells Migration and Invasion

**FIGURE 8:**
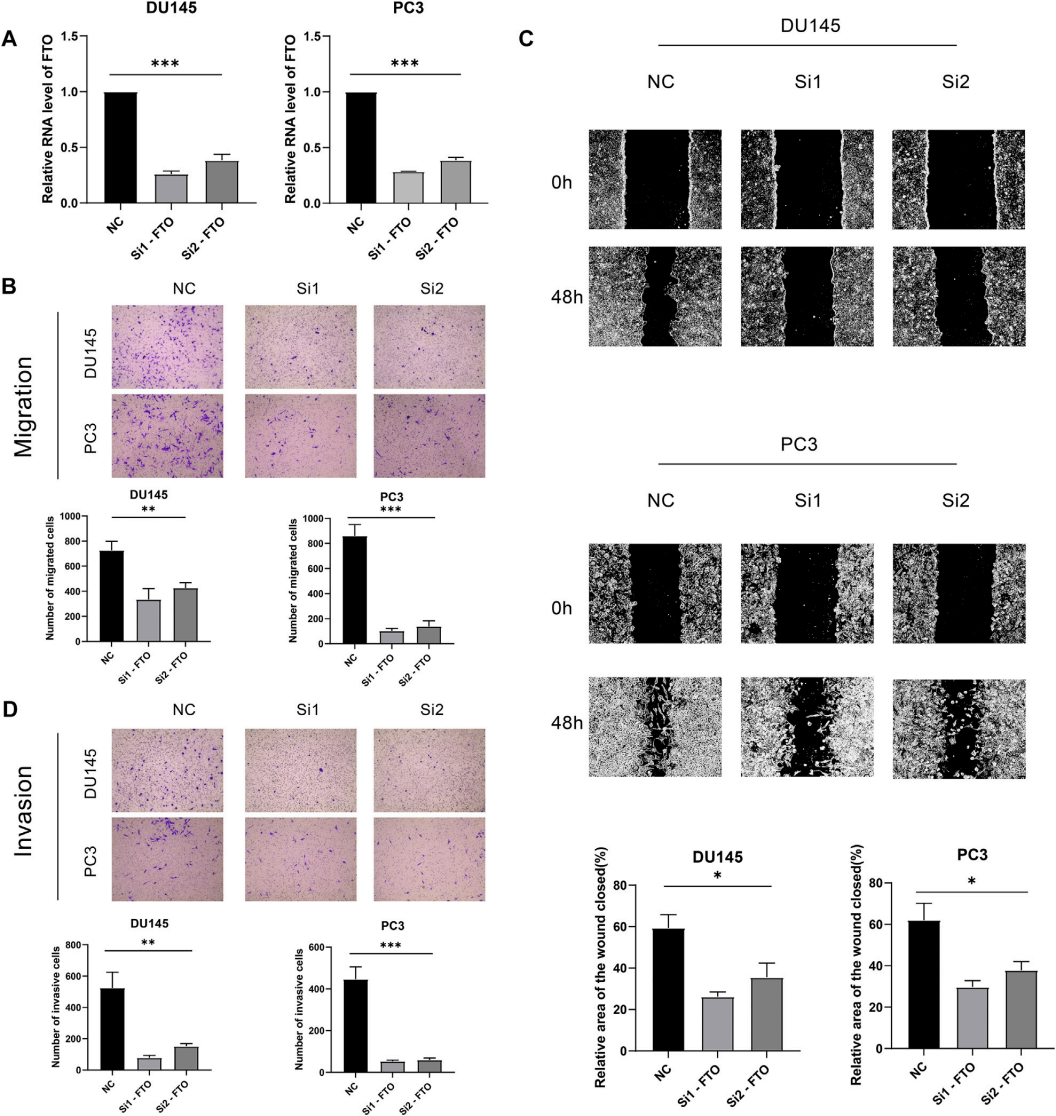
**(A)** Vitro experiments. **(B)** Transwell assay. **(C)** Wound-healing assay. **(D)** Transwell assay with Matrigel.

**FIGURE 9:**
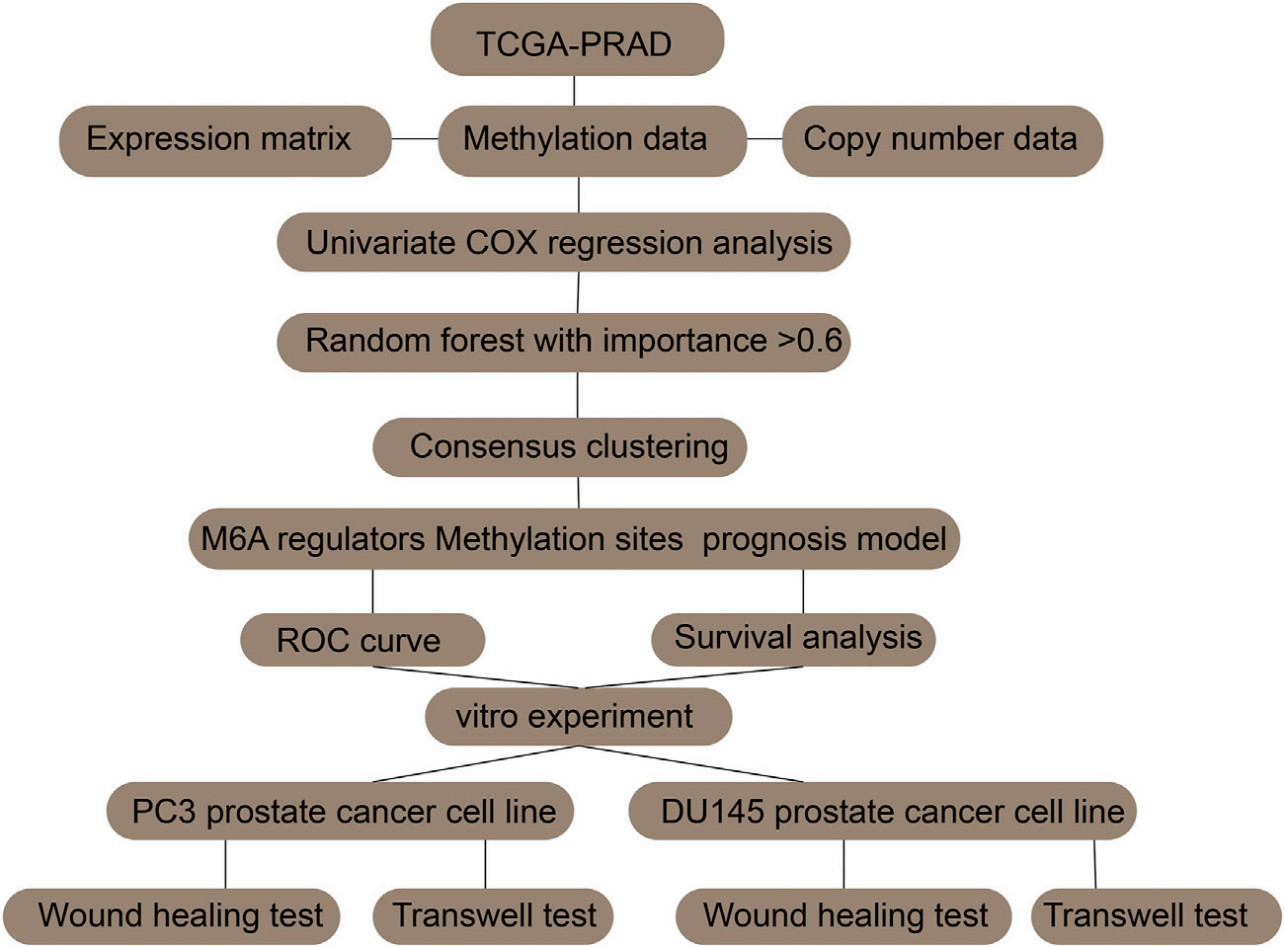
Flow chart.

Knockdown of FTO significantly inhibited prostate cancer cells migration and invasion to further explore the biological function of FTO in prostate cancer, a series of in vitro experiments were performed. Transwell assay was performed to measure the migration ability of FTOknockdown cells. The results indicated that the migration ability of cells in the FTOknockdown group was lower than that in the NC group (Figures 8A,B). The wound-healing assay was used to investigate the influence of FTO on the migration of prostate cancer cells. The results indicated that decreased FTO resulted in a decrease in the migration ability of prostate cancer cells (Figure 8C). Transwell assay with Matrigel indicated that the invasion ability of prostate cancer cells was decreased due to the knockdown of FTO (Figure 8D). The process of the results was summarized in Figure 9.

## DISCUSSION

Accumulating evidence shows that m6A methylation greatly impacts RNA metabolism and is related to the pathogenesis of numerous forms of illnesses, including a variety of cancers. This article primarily concentrates on the physiological functions of m6A modification as well as the m6A regulators involved in prostate cancer. Further, premised on the m6A regulators, prostate cancer was classified into three cohorts with different prognosis.

To date, many research reports have indicated abnormal expression of m6A regulators in prostate cancer. A few studies found that METTL3, an m6A writer, has high expression in PCa tissues and cells. METTL3 regulates various biological processes such as cell proliferation, cell migration and invasion, cell differentiation, apoptosis, and inflammatory response (Liu et al., 2020). Further, mechanism analysis showed that silencing the METTL3 gene might reduce the m6A modification as well as GLI1 expression. GLI1 is an essential element of the hedgehog pathway and has a vital function in cell apoptosis and PCa progression. In this regard, high METTL3 expression promoted the growth of PCa tissues *in vivo (* *Cai et al., 2019* *)*. Moreover, METTL3 over-expression is an adverse prognostic factor for DFS as well as OS among PCa patients. Mechanically, METTL3 enhances MYC (c-myc) expression by inducing an increase in m6A levels of MYC mRNA and affects the activity of the Wnt pathway through m6A methylation of LEF1 mRNA. These METTL3-induced changes promote the progression of PCa (Ma et al., 2020; Yuan et al., 2020). METTL3 can adjust the Integrin β1 (ITGB1) expression *via* the m6A-HuR-dependent mechanism. This could affect ITGB1 binding to type I collagen and tumor cell motility, which helps to promote bone metastasis in the PCa (Li et al., 2020). Additional studies also discovered that YTHDF2, an m6A reader, is up-regulated in PCa tissues and cells. Knockdown of YTHDF2 elevated m6A levels and inhibited the proliferative and migrative ability of PCa cell lines. In contrast, high YTHDF2 expression promotes the progression of PCa (Li et al., 2018). Other research showed that METTL3 and YTHDF2 were upregulated in PCa tissues. Tumor suppressors NKX3.1 and LHPP were the precise targets of METTL3 and YTHDF2. YTHDF2 has been known to mediate the mRNA degradation of NKX3.1 and LHPP in an m6A-dependent way to regulate AKT phosphorylation-induced prostate cancer progression (Li J. et al., 2020). On the other hand, studies found that FTO, an m6A eraser, was down-regulated in PCa tissues and cell lines. It was also noted that prostate cancer patients with decreased FTO expression often had high tumor stage and high Gleason scores. These studies reveal that FTO can impede the invasion and migration of prostate cancer cells through regulating the m6A levels (Zhu K. et al., 2021).

RNA m6A methylation provides a new regulatory mechanism for the mechanistic study of prostate cancer. In our study, we identified 27 m6A regulators, which exhibit aberrant gene expression in PCa. Further, we found that DNA methylation and CNVs of m6A regulators affect their expression and are involved in the tumorigenesis of prostate cancer. Besides, we discovered that DNA methylation levels, gene expression, and CNVs of several m6A regulators were correlated, either negatively or positively, with the disease outcome and prognosis, which could help with the precise treatment of prostate cancer. The levels of gene expression, CNVs, and DNA methylation are not at all times elevated, and they could have different or opposite prognostic values. The complex gene expression regulatory network may be the reason for this inconsistency.

Prostate cancer is a complex disease. Studies on gene expression have a crucial function in prolonging patients’ survival as a result of the improvement in accurate diagnosis and targeted treatment in recent years. Some researchers found 41 mRNA m6A regulators have been identified to play critical roles in primary prostate cancer. The translation initiation factor subunit EIF3D may delay the prostate cancer progression while the splicing factor HNRNPA2B1 may promote prostate cancer progression, *in vitro* assays proved the roles of EIF3D as well as HNRNPA2B1 in prostate cancer cells (Jiang et al., 2020). It was found that the expression of METTL14, IGF2BP3, HNRNPA2B1, and CNV of ALKBH5 were linked to the recurrence-free survival of prostate cancer. And m6A methylation can promote the progression of prostate cancer by ​regulating subcellular protein localization (Ji et al., 2020). Moreover, researchers constructed a survival prediction signature for PCa based on MRTTL14 and YTHDF2. They also established an interaction network of various m6A RNA regulators in PCa (Wang J. et al., 2020). Another research has shown that YTHDF1/2, YTHDC2, and METTL3 were upregulated in PCa, but METTL14, FTO, and ALKBH5 were down-regulated. In addition, the results found that YTHDF1, YTHDF2, and YTHDC2 exhibited a positive correlation, but METTL14, FTO, and ALKBH5 were negatively correlated to Gleason grades (Wu et al., 2021). Some researchers analyzed the important m6A RNA regulators, which act as prognosis factors in prostate cancer, and identified RBMX, NXF1, YTHDF1, HNRNPA2B1, and TRMT112 as critical genes that have great effects on the prognosis of PCa patients (Xu et al., 2020). Many studies have also explored expression patterns and clinical prognostic values of m6A regulators in PCa, which plays a great function in the treatment of PCa (Ou-Yang et al., 2020; Zhang et al., 2021). Nevertheless, in prostate cancer with different Gleason scores, there was still heterogeneity of PCa. In our study, we carried out M6A regulator genes methylation prognosis model to predict the prognosis of prostate cancer.

Effective biomarkers are very important in helping patients to get suitable treatment. There are also other studies to show the association between m6A and PCa. The nuclear-enriched abundant tran-script 1(NEAT1) acts as a long non-coding RNA(ncRNA) has high expression in a variety of cancer types. Studies found that high m6A level of NEAT1–1 was correlated with bone metastasis of prostate cancer as well as m6A level of NEAT1–1 may be new target for diagnosis and therapy of bone metastatic prostate cancer (Wen et al., 2020). A few studies revealed that immune cell infiltration has an imperative function in prostate cancer progression, which indicates the potential of personalized immunotherapy in prostate cancer patients (Zhao et al., 2021). Researchers have found that in many database(for example RMVar and RMDisease), m6A and other RNA modifications are closely related to the progress of various diseases (Chen et al., 2021; Luo et al., 2021). In our study, we conducted a study on the correlation between m6A and the progression and prognosis of prostate cancer in TCGA database to further supplement the relationship between m6A and diseases. Prostate cancers were divided into three different groups premised on DNA methylation levels of m6A regulators. Based on the expression of CBLL1, we also identified appropriate candidate agents with higher drug sensitivity for PCa, which is beneficial for the treatment of PCa.

HNRNPA2B1 is a member of HNRNP family, it is a pre mRNA binding protein, which is involved in mRNA localization, splicing, processing, stability and translation (Dreyfuss et al., 2002). HNRNPA2B1 also acts as the m6A “reader” and very crucial in the occurrence and development of many types of cancer. Studies found that HNRNPA2B1 was the most critically prognostic locus of m6A regulatory genes in oral squamous cell carcinoma(OSCC), silence of HNRNPA2B1 could inhibit the proliferation, migration, and invasion of OSCC by inducing epithelial-to-mesenchymal transition(EMT) (Zhu F. et al., 2021). Still other studies have found that HNRNPA2B1 can promote cell proliferation and regulate apoptosis of human colon cancer *via* the ERK/MAPK signaling, which indicates HNRNPA2B1 provides a potential treatment site for colon cancer (Tang et al., 2021). At the same time, also studies have found that HNRNPA2B1 was the oncogenic gene in esophageal cancer(ESCA), the increased expression of HNRNPA2B1 could predict adverse prognosis of ESCA by affecting tumor-promoting signaling pathways, silencing HNRNPA2B1 could inhibit the proliferation of ESCA cells (Li et al., 2021). Also overexpression of HNRNPA2B1 is correlated with poor survival in gastric cancer (GC), multiple myeloma (MM) and ovarian cancer and HNRNPA2B1 acts as an oncogenic role in their progression (Yang et al., 2020; Jiang et al., 2021; Peng et al., 2021).

In our study, we investigated the role of FTO in the proliferation of the prostate cancer cell. We found that FTO was down regulated in prostate cancers. Knockdown of FTO prominently inhibited prostate cells migration as well as invasion. So our study highlights that FTO is an oncogenic gene that promotes the progression of prostate cancer, and it is a potential novel therapeutic target for treatment of prostate cancer.

In summary, our study carried out a multi-omics comprehensive analysis in prostate cancer to identify the m6A regulators. Further, premised on DNA methylation of m6A regulators, prostate cancers were divided into three different subgoups, which were associated with different disease-free survival. Subsequently, we found that Knockdown of FTO prominently inhibited prostate cells migration and invasion. Our study helps to explore the mechanism of prostate cancer and develop new strategies for personalized treatment of prostate cancer.

## Data Availability

The datasets presented in this study can be found in online repositories. The names of the repository/repositories and accession number(s) can be found in the article/Supplementary Material.

## References

[B2] Cai J. Yang F. Zhan H. Situ J. Li W. Mao Y. (2019). RNA m6A Methyltransferase METTL3 Promotes the Growth of Prostate Cancer by Regulating Hedgehog Pathway. Onco Targets Ther. 12, 9143–9152. 10.2147/ott.s226796 31806999 PMC6842310

[B3] Chen K. Song B. Tang Y. Wei Z. Xu Q. Su J. (2021). RMDisease: a Database of Genetic Variants that Affect RNA Modifications, with Implications for Epitranscriptome Pathogenesis. Nucleic Acids Res. 49, D1396–d1404. 10.1093/nar/gkaa790 33010174 PMC7778951

[B4] Chen X.-Y. Zhang J. Zhu J.-S. (2019). The Role of m6A RNA Methylation in Human Cancer. Mol. Cancer 18, 103. 10.1186/s12943-019-1033-z 31142332 PMC6540575

[B5] Chou C.-H. Shrestha S. Yang C.-D. Chang N.-W. Lin Y.-L. Liao K.-W. (2018). miRTarBase Update 2018: a Resource for Experimentally Validated microRNA-Target Interactions. Nucleic Acids Res. 46, D296–d302. 10.1093/nar/gkx1067 29126174 PMC5753222

[B6] Diaz-Uriarte R. (2007). GeneSrF and varSelRF: a Web-Based Tool and R Package for Gene Selection and Classification Using Random forest. BMC Bioinformatics 8, 328. 10.1186/1471-2105-8-328 17767709 PMC2034606

[B7] Dreyfuss G. Kim V. N. Kataoka N. (2002). Messenger-RNA-binding Proteins and the Messages They Carry. Nat. Rev. Mol. Cel Biol 3, 195–205. 10.1038/nrm760 11994740

[B8] Egevad L. Delahunt B. Yaxley J. Samaratunga H. (2019). Evolution, Controversies and the Future of Prostate Cancer Grading. Pathol. Int. 69, 55–66. 10.1111/pin.12761 30694570

[B9] Epstein J. I. Egevad L. Amin M. B. Delahunt B. Srigley J. R. Humphrey P. A. (2016a). The 2014 International Society of Urological Pathology (ISUP) Consensus Conference on Gleason Grading of Prostatic Carcinoma. Am. J. Surg. Pathol. 40, 244–252. 10.1097/pas.0000000000000530 26492179

[B10] Epstein J. I. Zelefsky M. J. Sjoberg D. D. Nelson J. B. Egevad L. Magi-Galluzzi C. (2016b). A Contemporary Prostate Cancer Grading System: A Validated Alternative to the Gleason Score. Eur. Urol. 69, 428–435. 10.1016/j.eururo.2015.06.046 26166626 PMC5002992

[B11] Ferlay J. Soerjomataram I. Dikshit R. Eser S. Mathers C. Rebelo M. (2015). Cancer Incidence and Mortality Worldwide: Sources, Methods and Major Patterns in GLOBOCAN 2012. Int. J. Cancer 136, E359–E386. 10.1002/ijc.29210 25220842

[B12] Goldman M. J. Craft B. Hastie M. Repečka K. Mcdade F. Kamath A. (2020). Visualizing and Interpreting Cancer Genomics Data via the Xena Platform. Nat. Biotechnol. 38, 675–678. 10.1038/s41587-020-0546-8 32444850 PMC7386072

[B13] Haunsberger S. J. Connolly N. M. Prehn J. H. (2017). miRNAmeConverter: an R/bioconductor Package for Translating Mature miRNA Names to Different miRBase Versions. Bioinformatics 33, 592–593. 10.1093/bioinformatics/btw660 27797767

[B14] Ji G. Huang C. He S. Gong Y. Song G. Li X. (2020). Comprehensive Analysis of m6A Regulators Prognostic Value in Prostate Cancer. Aging 12, 14863–14884. 10.18632/aging.103549 32710725 PMC7425456

[B15] Jiang F. Tang X. Tang C. Hua Z. Ke M. Wang C. (2021). HNRNPA2B1 Promotes Multiple Myeloma Progression by Increasing AKT3 Expression via m6A-dependent Stabilization of ILF3 mRNA. J. Hematol. Oncol. 14, 54. 10.1186/s13045-021-01066-6 33794982 PMC8017865

[B16] Jiang M. Lu Y. Duan D. Wang H. Man G. Kang C. (2020). Systematic Investigation of mRNA N 6-Methyladenosine Machinery in Primary Prostate Cancer. Dis. Markers 2020, 8833438. 10.1155/2020/8833438 33273988 PMC7676945

[B1] Kimura S. Tokuhisa M. Okada M. (2019). Inference of Genetic Networks Using Random Forests: Assigning Different Weights for Gene Expression Data. J. Bioinform. Comput. Biol. 17 (4), 1950015. 10.1142/s021972001950015x 31291807

[B17] Li E. Wei B. Wang X. Kang R. (2020). METTL3 Enhances Cell Adhesion through Stabilizing Integrin β1 mRNA via an m6A-HuR-dependent Mechanism in Prostatic Carcinoma. Am. J. Cancer Res. 10, 1012–1025. 32266107 PMC7136910

[B18] Li J. Meng S. Xu M. Wang S. He L. Xu X. (2018). Downregulation of N6-Methyladenosine Binding YTHDF2 Protein Mediated by miR-493-3p Suppresses Prostate Cancer by Elevating N6-Methyladenosine Levels. Oncotarget 9, 3752–3764. 10.18632/oncotarget.23365 29423080 PMC5790497

[B19] Li J. Xie H. Ying Y. Chen H. Yan H. He L. (2020a). YTHDF2 Mediates the mRNA Degradation of the Tumor Suppressors to Induce AKT Phosphorylation in N6-methyladenosine-dependent Way in Prostate Cancer. Mol. Cancer 19, 152. 10.1186/s12943-020-01267-6 33121495 PMC7599101

[B20] Li Y. Ge Y.-z. Xu L. Xu Z. Dou Q. Jia R. (2020b). The Potential Roles of RNA N6-Methyladenosine in Urological Tumors. Front. Cel Dev. Biol. 8, 579919. 10.3389/fcell.2020.579919 PMC7510505 33015074

[B21] Li K. Chen J. Lou X. Li Y. Qian B. Xu D. (2021). HNRNPA2B1 Affects the Prognosis of Esophageal Cancer by Regulating the miR-17-92 Cluster. Front. Cel Dev. Biol. 9, 658642. 10.3389/fcell.2021.658642 PMC8278577 34277606

[B22] Liu S. Zhuo L. Wang J. Zhang Q. Li Q. Li G. (2020). METTL3 Plays Multiple Functions in Biological Processes. Am. J. Cancer Res. 10, 1631–1646. 32642280 PMC7339281

[B23] Love M. I. Huber W. Anders S. (2014). Moderated Estimation of Fold Change and Dispersion for RNA-Seq Data with DESeq2. Genome Biol. 15, 550. 10.1186/s13059-014-0550-8 25516281 PMC4302049

[B24] Luo J. Liu H. Luan S. He C. Li Z. (2018). Aberrant Regulation of mRNA m⁶A Modification in Cancer Development. Int. J. Mol. Sci. 19, 2515. 10.3390/ijms19092515 PMC6164065 30149601

[B25] Luo X. Li H. Liang J. Zhao Q. Xie Y. Ren J. (2021). RMVar: an Updated Database of Functional Variants Involved in RNA Modifications. Nucleic Acids Res. 49, D1405–d1412. 10.1093/nar/gkaa811 33021671 PMC7779057

[B26] Ma X. X. Cao Z. G. Zhao S. L. (2020). m6A Methyltransferase METTL3 Promotes the Progression of Prostate Cancer via m6A-Modified LEF1. Eur. Rev. Med. Pharmacol. Sci. 24, 3565–3571. 10.26355/eurrev_202004_20817 32329830

[B27] Martínez-Breijo S. Chantada-Abal V. Aller-Rodríguez M. Bohórquez-Cruz M. Sacristán-Lista F. Ponce-Díaz J. (2018). Castration Resistance Mechanisms in Prostate Cancer. Arch. Esp Urol. 71, 628–638. 30319123

[B28] Mcnevin C. S. Baird A. M. Mcdermott R. Finn S. P. (2021). Diagnostic Strategies for Treatment Selection in Advanced Prostate Cancer. Diagnostics (Basel) 11, 345. 10.3390/diagnostics11020345 33669657 PMC7922176

[B29] Mermel C. H. Schumacher S. E. Hill B. Meyerson M. L. Beroukhim R. Getz G. (2011). GISTIC2.0 Facilitates Sensitive and Confident Localization of the Targets of Focal Somatic Copy-Number Alteration in Human Cancers. Genome Biol. 12, R41. 10.1186/gb-2011-12-4-r41 21527027 PMC3218867

[B30] Ou-Yang S. Liu J. H. Wang Q. Z. (2020). Expression Patterns and a Prognostic Model of m6A-Associated Regulators in Prostate Adenocarcinoma. Biomark Med. 14, 1663–1677. 10.2217/bmm-2020-0095 33336591

[B31] Peng W.-z. Zhao J. Liu X. Li C.-f. Si S. Ma R. (2021). hnRNPA2B1 Regulates the Alternative Splicing of BIRC5 to Promote Gastric Cancer Progression. Cancer Cel Int 21, 281. 10.1186/s12935-021-01968-y PMC8161968 34044823

[B32] Sarkar K. Chowdhury R. Dasgupta A. (2017). Analysis of Survival Data: Challenges and Algorithm-Based Model Selection. J. Clin. Diagn. Res. 11, Lc14–lc20. 10.7860/JCDR/2017/21903.10019 PMC5532537 28764206

[B33] Siegel R. L. Miller K. D. Fuchs H. E. Jemal A. (2021). Cancer Statistics, 2021. CA A. Cancer J. Clin. 71, 7–33. 10.3322/caac.21654 33433946

[B34] Sinnott J. A. Cai T. (2016). Inference for Survival Prediction under the Regularized Cox Model. Biostat 17, 692–707. 10.1093/biostatistics/kxw016 PMC5031946 27107008

[B35] Tang J. Chen Z. Wang Q. Hao W. Gao W.-Q. Xu H. (2021). hnRNPA2B1 Promotes Colon Cancer Progression via the MAPK Pathway. Front. Genet. 12, 666451. 10.3389/fgene.2021.666451 34630502 PMC8494201

[B36] Wang J. Lin H. Zhou M. Xiang Q. Deng Y. Luo L. (2020a). The m6A Methylation Regulator-Based Signature for Predicting the Prognosis of Prostate Cancer. Future Oncol. 16, 2421–2432. 10.2217/fon-2020-0330 32687727

[B37] Wang T. Kong S. Tao M. Ju S. (2020b). The Potential Role of RNA N6-Methyladenosine in Cancer Progression. Mol. Cancer 19, 88. 10.1186/s12943-020-01204-7 32398132 PMC7216508

[B38] Wen S. Wei Y. Zen C. Xiong W. Niu Y. Zhao Y. (2020). Long Non-coding RNA NEAT1 Promotes Bone Metastasis of Prostate Cancer through N6-Methyladenosine. Mol. Cancer 19, 171. 10.1186/s12943-020-01293-4 33308223 PMC7733260

[B39] Wilkerson M. D. Hayes D. N. (2010). ConsensusClusterPlus: a Class Discovery Tool with Confidence Assessments and Item Tracking. Bioinformatics 26, 1572–1573. 10.1093/bioinformatics/btq170 20427518 PMC2881355

[B40] Wu Q. Xie X. Huang Y. Meng S. Li Y. Wang H. (2021). N6-methyladenosine RNA Methylation Regulators Contribute to the Progression of Prostate Cancer. J. Cancer 12, 682–692. 10.7150/jca.46379 33403026 PMC7778550

[B41] Xu J. Liu Y. Liu J. Xu T. Cheng G. Shou Y. (2020). The Identification of Critical m6A RNA Methylation Regulators as Malignant Prognosis Factors in Prostate Adenocarcinoma. Front. Genet. 11, 602485. 10.3389/fgene.2020.602485 33343639 PMC7746824

[B42] Yang Y. Wei Q. Tang Y. Wang Y. Luo Q. Zhao H. (2020). Loss of hnRNPA2B1 Inhibits Malignant Capability and Promotes Apoptosis via Down-Regulating Lin28B Expression in Ovarian Cancer. Cancer Lett. 475, 43–52. 10.1016/j.canlet.2020.01.029 32006618

[B43] Yu G. Wang L.-G. Han Y. He Q.-Y. (2012). clusterProfiler: an R Package for Comparing Biological Themes Among Gene Clusters. OMICS: A J. Integr. Biol. 16, 284–287. 10.1089/omi.2011.0118 PMC3339379 22455463

[B44] Yuan Y. Du Y. Wang L. Liu X. (2020). The M6A Methyltransferase METTL3 Promotes the Development and Progression of Prostate Carcinoma via Mediating MYC Methylation. J. Cancer 11, 3588–3595. 10.7150/jca.42338 32284755 PMC7150444

[B45] Zhang Q. Luan J. Song L. Wei X. Xia J. Song N. (2021). Malignant Evaluation and Clinical Prognostic Values of M6A RNA Methylation Regulators in Prostate Cancer. J. Cancer 12, 3575–3586. 10.7150/jca.55140 33995635 PMC8120168

[B46] Zhao B. S. Roundtree I. A. He C. (2017). Post-transcriptional Gene Regulation by mRNA Modifications. Nat. Rev. Mol. Cel Biol 18, 31–42. 10.1038/nrm.2016.132 PMC5167638 27808276

[B47] Zhao Y. Sun H. Zheng J. Shao C. (2021). Analysis of RNA m6A Methylation Regulators and Tumour Immune Cell Infiltration Characterization in Prostate Cancer. Artif. Cell Nanomedicine, Biotechnol. 49, 407–435. 10.1080/21691401.2021.1912759 33905280

[B48] Zheng H.-x. Zhang X.-s. Sui N. (2020). Advances in the Profiling of N6-Methyladenosine (m6A) Modifications. Biotechnol. Adv. 45, 107656. 10.1016/j.biotechadv.2020.107656 33181242

[B49] Zhu F. Yang T. Yao M. Shen T. Fang C. (2021). HNRNPA2B1, as a m6A Reader, Promotes Tumorigenesis and Metastasis of Oral Squamous Cell Carcinoma. Front. Oncol. 11, 716921. 10.3389/fonc.2021.716921 34631545 PMC8494978

[B50] Zhu K. Li Y. Xu Y. (2021). The FTO m6A Demethylase Inhibits the Invasion and Migration of Prostate Cancer Cells by Regulating Total m6A Levels. Life Sci. 271, 119180. 10.1016/j.lfs.2021.119180 33571513

