# Corrigendum: RNA m6A Methylation Regulators Multi-Omics Analysis in Prostate Cancer

**DOI:** 10.3389/fgene.2021.845413

**Published:** 2022-01-18

**Authors:** Hao Su, Yutao Wang, Hongjun Li

**Affiliations:** ^1^ Department of Urology, Chinese Academy of Medical Sciences, Peking Union Medical College, Peking Union Medical College Hospital, Beijing, China; ^2^ Department of Urology, The First Affiliated Hospital of China Medical University, Shenyang, China

**Keywords:** m6A methylation, methylation prognosis model, muti-omics analysis, prostate cancer, vitro experiment

In the original article, there was an error. “FTO is misspelled as HNRNPA2B1 on six occasions.”

A correction has been made to **Abstract**, **Paragraph 1**:

“Premised on the expression of CBLL1, we also identified potential therapeutic agents for prostate cancer, and knockdown of FTO prominently inhibited prostate cells migration and invasion *in vitro* experiment.”

A correction has been made to **Materials and Methods**, **Cell Culture and Transfection**, Paragraph 1:

“The two Small interfering RNAs (JTSBIO) sequences used to reduce FTO expression sequences used to reduce FTO expression were as follows: GCA​GUG​UAU​CUG​AGG​AGC​UCC​AUA​A UUA​UGG​AGC​UCC​UCA​GAU​ACA​CUG​C; CAG​GCU​GCA​CCU​ACA​AGU​ACC​UGA​A UUC​AGG​UAC​UUG​UAG​GUG​CAG​CCU​G.”

A correction has been made to **Materials and Methods**, **RNA Extraction and Quantitative Real-Time PCR**, Paragraph 1:

“RT-qPCR was performed using SYBR Premix EX Taq™ (Takara) and 2^−ΔΔCT^ method was used to analyze the expression level of FTO normalized to GAPDH. The primer sequences are as follows: FTO (forward: ACT​TGG​CTC​CCT​TAT​CTG​ACC; reverse: TGT​GCA​GTG​TGA​GAA​AGG​CTT) and GAPDH (forward: GGA​GCG​AGA​TCC​CTC​CAA​AAT; reverse: GGC​TGT​TGT​CAT​ACT​TCT​CAT​GG).”

A correction has been made to **Results**, **Knockdown of HNRNPA2B1 Significantly Inhibited Prostate Cells Migration and Invasion**, Paragraph 1:

“Knockdown of FTO significantly inhibited prostate cancer cells migration and invasion to further explore the biological function of FTO in prostate cancer, a series of *in vitro* experiments were performed. Transwell assay was performed to measure the migration ability of FTO-knockdown cells. The results indicated that the migration ability of cells in the FTO-knockdown group was lower than that in the NC group (**Figures 8A,B**). The wound-healing assay was used to investigate the influence of FTO on the migration of prostate cancer cells. The results indicated that decreased FTO resulted in a decrease in the migration ability of prostate cancer cells (**Figure 8C**). Transwell assay with Matrigel indicated that the invasion ability of prostate cancer cells was decreased due to the knockdown of FTO (**Figure 8D**). The process of the results was summarized in **Figure 9**.”

A correction has been made to **Discussion**, Paragraph 7:

“In our study, we investigated the role of FTO in the proliferation of the prostate cancer cell. We found that FTO was down regulated in prostate cancers. Knockdown of FTO prominently inhibited prostate cells migration as well as invasion. So our study highlights that FTO is an oncogenic gene that promotes the progression of prostate cancer, and it is a potential novel therapeutic target for treatment of prostate cancer.”

A correction has been made to **Discussion**, Paragraph 8:

“In summary, our study carried out a multi-omics comprehensive analysis in prostate cancer to identify the m6A regulators. Further, premised on DNA methylation of m6A regulators, prostate cancers were divided into three different subgoups, which were associated with different disease-free survival. Subsequently, we found that Knockdown of FTO prominently inhibited prostate cells migration and invasion. Our study helps to explore the mechanism of prostate cancer and develop new strategies for personalized treatment of prostate cancer.”

The authors apologize for this error and state that this does not change the scientific conclusions of the article in any way. The original article has been updated.

